# Early *Versus* Delayed Enteral Feeding in Predicted Severe Acute Gallstone Pancreatitis: A Retrospective Study

**DOI:** 10.3389/fcimb.2022.938581

**Published:** 2022-07-22

**Authors:** Zheng Jin, Yaping Wei, Shanshan Hu, Minhui Sun, Mengdie Fang, Hongzhang Shen, Jianfeng Yang, Xiaofeng Zhang, Hangbin Jin

**Affiliations:** Affiliated Hangzhou First People’s Hospital, Zhejiang University School of Medicine, Hangzhou, China

**Keywords:** pancreatitis, enteral nutrition, infection, mortality, multiple drug resistant bacteria

## Abstract

**Background:**

The optimal timing of enteral nutrition (EN) initiation in predicted severe acute gallstone pancreatitis (SAGP) and its influence on disease outcomes are not well known.

**Methods:**

We conducted a retrospective study of patients with predicted SAGP treated with endoscopic retrograde cholangiopancreatography and EN. The patients were classified into two groups according to the timing of EN initiation after admission: within 48 h, and more than 48 h. The primary outcome was in-hospital mortality. The secondary outcomes were length of hospital stay, need for intensive care admission, need for surgical intervention, improvements in blood test results after 7-10 days of EN, incidence of pancreatic necrosis and infection, and hospital care costs. The microbiological profiles of infectious complications were also evaluated.

**Results:**

Of the 98 patients, 31 and 67 started EN within 48 h, and more than 48 h after admission, respectively. Early EN was associated with a decrease in in-hospital mortality (0 vs. 11.9%; p=0.045), length of hospital stay (median:18 *vs*. 27 days; p=0.001), need for intensive care admission (3.2% *vs*. 20.9%; p=0.032), and hospital care costs (median:9,289 *vs*. 13,518 US$; p=0.007), compared to delayed EN. Moreover, early EN for 7-10 days had more beneficial effects on blood test results than delayed EN, including total protein (p=0.03) and CRP (p=0.006) levels. However, the need for surgical intervention and incidence of pancreatic necrosis did not differ between the two groups. In our study, Gram-negative bacteria were the main responsible pathogens (50.5%). Infection with multidrug-resistant organisms (MDRO) was found in 19.4% of the patients. The most common MDRO was MDR *Enterococcus faecium*. Early EN was not superior in reducing incidence of infected pancreatic necrosis, bacteremia, polymicrobial infection, or MDROs.

**Conclusions:**

In patients with predicted SAGP, early EN is associated with a decrease in in-hospital mortality, length of hospital stay, need of intensive care admission, and hospital care costs, compared to delayed EN. There are no significant benefits of early EN in reducing the rate of infection-related complications. Further studies with larger sample sizes are warranted.

## Introduction

Acute pancreatitis (AP) is the most common pancreatic disease in the world. Severe acute pancreatitis (SAP) develops in approximately 20–30% of patients with AP and is associated with a high mortality rate of up to 30%. (Leppaniemi et al., 2019) Gallstones are the most common cause of AP. (Yadav and Lowenfels, 2013) When accompanying biliary obstruction occurs, patients with gallstone pancreatitis are recommended to undergo endoscopic retrograde cholangiopancreatography (ERCP) to remove obstructing gallstones, in order to ameliorate the disease course. (Arvanitakis et al., 2018) The traditional approach for treating AP patients has been based on bowel rest. However, current guidelines recommend early (generally within 24 h) enteral nutrition (EN) for AP, to maintain the gut mucosal barrier and prevent infected necrosis. (Crockett et al., 2018) Nonetheless, there is still a need to more precisely define the optimal timing for initiating early EN in patients with predicted severe acute gallstone pancreatitis (SAGP). Moreover, knowledge on the effect of early EN on the bacteria/fungi spectrum and antibiotic resistance characteristics of AP is limited.

Therefore, we conducted a single-center retrospective study in patients with SAGP, comparing the efficacy of early EN (within 48 h of admission) with delayed EN (after more than 48 h of admission), and further investigated the microbiological profiles of infectious complications associated with different routes of nutritional support.

## Materials and Methods

### Study Design and Participants

A retrospective analysis was performed on consecutive patients diagnosed with predicted SAGP and treated with ERCP and EN from March 2012 to March 2022 in a tertiary care hospital. The exclusion criteria were as follows: age ≤ 18 years; pregnancy; pancreatitis due to other causes such as hyperlipemia, alcohol abuse, chronic pancreatitis, etc.; patients who did not receive ERCP and/or EN; patients presenting to the hospital more than four days after symptom onset; or no data on any of the primary or secondary outcomes.

We defined EN initiation within 48 h of admission as early EN, and initiation more than 48 h of admission as delayed EN. AP was diagnosed as the presence of at least two of the following criteria: typical abdominal pain, a serum lipase or amylase level that was more than three times the upper limit of normal (ULN), or characteristic findings on imaging. (Banks et al., 2012) Gallstone pancreatitis was diagnosed by either biliary sludge or gallstones on imaging, a dilated common bile duct on imaging, or an alanine aminotransferase concentration of more than twice the ULN. (Tenner et al., 2013) Predicted SAP was defined based on an Acute Physiology and Chronic Health Evaluation (APACHE-II) score of eight or more, a Bedside Index of Severity in AP (BISAP) score of two or more, a computed tomography severity index (CTSI) score of three or more, or serum C-reactive protein concentration higher than 150 mg/L within 24 h of admission. (Papachristou et al., 2010; Cho et al., 2015) System Inflammatory Reaction Syndrome (SIRS) was defined as the presence of at least two of the following parameters for a continuous period of 48 h: body temperature > 38°C or < 36°C heart rate > 90 beats per minute; hyperventilation with a respiratory rate > 20 breath per minute or a PaCO_2_ < 32 mmHg; and white blood cell count > 12,000/mm^3^ or <4000/mm^3^. (Bone et al., 1992) Organ failure was defined as a modified Marshall score of 2 or more as proposed in the revised Atlanta classification of acute pancreatitis. (Banks et al., 2012) Cholestasis was defined as a serum bilirubin of more than 2.3 mg/dL (40 μmol/L) or a dilated common bile duct (> 8 mm in patients aged ≤ 75 years, or >10 mm in patients aged > 75 years). (Schepers et al., 2020) Cholangitis was defined as fever in combination with either common bile duct stones, a dilated common bile duct, or (progressive) cholestasis. (Schepers et al., 2020) Isolation of pathogens was defined as a positive culture obtained from blood, and/or the drainage of percutaneous, endoscopic procedure, or surgery.

### Procedures

In the present study, ERCP was performed within 48 h of admission. Considering that some patients had two or more ERCP procedures during their hospitalization, we only evaluated the first ERCP session. Biliary sphincterotomy was performed when the presence of bile duct stones was confirmed. If the common bile duct could not be cannulated, precut sphincterotomy was performed. Stones or sludge were extracted using baskets and/or balloons, if indicated. Stents or nasobiliary tubes were placed at the discretion of endoscopists. A nasoenteral feeding tube was placed endoscopically. According to the timing of EN initiation, patients were classified into one of two groups: early EN group, in which the nasoenteral feeding tube was placed in the same session with ERCP, and EN was initiated within 48 h of admission; and delayed EN group, in which the timing of both nasoenteral feeding tube placement and EN initiation was more than 48 h of admission. Enteral nutritional suspension Peptison (NUTRICIA, Wuxi, China) was preheated to 100 degrees Fahrenheit and then initiated at a speed of 25 ml/h; the speed was increased by 10 ml/h every 6 h if tolerated, until the desired caloric intake of patients was reached. Oral feeding was reintroduced when the serum amylase level decreased to < 2-fold ULN and abdominal pain had resolved. Along with ERCP and EN, the patients were treated with standard medical therapy for AP, fluid resuscitation, and other supportive therapies for organ failure. Antibiotics were prescribed only if bacterial infection was confirmed. Third generation cephalosporins were initially used as antibiotics. Samples from blood, bile, or peri-pancreatic fluid were gram- and fluorescence -stained to identify pathogens. Isolated pathogens were tested for antimicrobial susceptibility using the VITEK2 compact automated microbiology system (BioMerienx, France), based on the standards of the Clinical and Laboratory Standards Institute. (Humphries et al., 2018)

### Data Collection

The following data were obtained using data collection sheets from electronic medical records: age, sex, body-mass index, evidence of gallstone etiology (such as gallstones or sludge, cholestasis, or cholangitis), and disease severity (such as APACHE-II score, CTSI score, BISAP score, and presence of SIRS or organ failure) within 12 h of admission; the time from pancreatitis onset to admission, the time from admission to ERCP, the time from admission to initiation of EN, and the time from initiation of EN to initiation of oral feeding; laboratory data including serum levels of total bilirubin, total protein, urea, white blood cell, procalcitonin and C-reactive protein (CRP) within 12 h of admission and after 7-10 days of EN; characteristics of pathogens isolated during hospitalization based on a blood culture, bile culture, or local culture; and radiological data including characteristics of contrast enhanced CT scan within 12 h of admission and after 7-10 days of EN, and characteristics of first ERCP after admission.

### Outcomes

The primary outcome was in-hospital mortality. Secondary outcomes were the length of hospital stay, need for intensive care admission, need for surgical intervention, improvements in blood test results after 7-10 days of EN (negative value indicated that the value of the result became larger after treatment), incidence of pancreatic necrosis, infected pancreatic necrosis, bacteremia, polymicrobial infection, multidrug-resistant organism (MDRO), and hospital care costs. Pancreatic necrosis was defined as the presence of diffuse or focal areas of pancreatic non-enhancement on contrast enhanced CT. (Banks et al., 2012) Infected pancreatic necrosis was diagnosed when positive culture from peripancreatic fluid collection was demonstrated, or the presence of gas within the collection was seen on CT. (Banks et al., 2012) Bacteremia was defined as an infection with positive blood cultures. Surgical interventions included endoscopic, laparoscopic, or laparotomy for drainage or necrosectomy in infected pancreatic necrosis. MDRO was defined as acquired non-susceptibility to at least one agent in three or more antimicrobial categories. (Magiorakos et al., 2012)

### Statistical Analysis

IBM SPSS Statistics version 22 was used for statistical analysis. Categorical variables were expressed as frequencies and percentages. The means and standard deviation were used for quantitative variables with parametric distribution, and the medians and interquartile range (IQR) were used for variables with nonparametric distributions. Dichotomous data were compared using the Pearson’s χ² test or Fisher’s exact test, and continuous data with the Student’s t-test for those with parametric distribution and the Mann-Whitney U test were used for those with non-parametric distribution. A two-sided p value of less than 0.05 was considered to indicate statistical significance.

## Results

### Baseline Characteristics of Patients

A total of 98 patients with a predicted SAGP were finally included, of which 31 (31.6%) were in the early EN group and 67 (68.4%) were in the delayed EN group. Baseline characteristics, presented in **Table 1**, were equally distributed between the two groups, except for the time from admission to ERCP (median [IQR]:13 [3-25] *vs*. 27 [9-64] h; p<0.05), and the time from admission to initiation of EN (median [IQR]:30 [15-33] *vs*. 128 [82-216] h; p=0.035). The time from pancreatitis onset to admission (median [IQR]:2 [2-3] *vs*. 2 [1-3] days; p=0.84) and the time from initiation of EN to initiation of oral feeding (mean [SD]:15 [14] *vs*. 16 [20] days; p=0.92) were similar between the two groups.

**Table 1:** Baseline demographic and clinical characteristics of patients.

	Early EN (n=31)	Delayed EN (n=67)	p Value
Age, yrs	71 (50-79)	65 (57-71)	0.65
Female sex	12 (38.7%)	33 (49.3%)	0.33
Basis of gallstone etiology^†^			0.58
Gallstones or sludge	24 (77.4%)	53 (79.1%)	
Cholestasis	13 (41.9%)	38 (56.7%)	
Cholangitis	20 (64.5%)	36 (53.7%)	
Body-mass index, kg/m²	23.7 (4.1)	24.8 (3.4)	0.32
Disease severity			
APACHE-II	7.2 (2.9)	7.2 (2.9)	0.52
CTSI	2.2 (1.1)	3.3 (2.4)	0.11
BISAP	2.2 (0.8)	2.3 (0.8)	0.61
CRP, mg/L	169.0 (104.9-264.4)	141.4 (62.2-202.8)	0.07
SIRS	19 (61.3%)	37 (55.2%)	0.57
Organ failure	7 (22.6%)	16 (23.9%)	0.89
Tests on admission to hospital			
Total bilirubin, μmol/L	28.6 (15.8-40.3)	31.7 (17.8-54.7)	0.27
Total protein, g/L	62.0 (55.5-67.9)	63.2 (59.1-70.1)	0.14
Urea, mmol/L	5.19 (3.6-6.8)	6.5 (5.0-8.2)	0.14
White blood cell, 10^3^/μL	15.2 (11.7-17.3)	12.6 (9.7-15.8)	0.17
Procalcitonin, ng/ml	5.33 (17.3)	5.26 (11.4)	0.62
Time from pancreatitis onset to admission, days	2 (2-3)	2 (1-3)	0.84
Time from admission to first ERCP, hrs	13 (3-25)	27 (9-64)	**<0.05**
Time from admission to initiation of EN, hrs	30 (15-33)	128 (82-216)	**0.035**
Time from initiation of EN to initiation of oral feeding, days	15 (14)	16 (20)	0.92

Data expressed as n (%), mean (SD), or median (IQR).

The bold values indicate that there was a significant difference.

APACHE, acute physiology and chronic health evaluation; BISAP, Bedside Index of Severity in acute pancreatitis; CRP, C-reactive protein; CTSI, computed tomography severity index; EN, enteral nutrition; ERCP, endoscopic retrograde cholangiopancreatography; SIRS, systemic inflammatory response syndrome.

^†^One case may involve one or more gallstone etiology.

### Clinical Outcomes

**Table 2** reports the clinical outcomes between the two groups. The overall in-hospital mortality was 8.2% (8/98). All deaths occurred in patients with delayed EN, and a significant difference was noted between the two groups (p=0.045). All patients that died were in the intensive care unit because of multiple organ failure. Early EN was associated with a shorter length of hospital stay (median [IQR]: 18 [12-30] *vs*. 27 [18-43] days; p=0.001) and a lower rate of intensive care admission (3.2% *vs*. 20.9%; p=0.032) than delayed EN. The need for surgical intervention (0% *vs*. 7.5%; p=0.18) and pancreatic necrosis incidence (87.0% *vs*. 91.0%; p=0.72) were similar between the two groups. After 7-10 days of EN support, there was a more significant improvement in total protein (median [IQR]: -3.9 [-9.2-1.3] *vs*. 2.1 [-5.4-6.1] g/L; p=0.03) and CRP (median [IQR]: 129.0 [63.8-177.4] *vs*.31.7 [-6.1-123.8] g/L; p=0.006) levels in patients from the early EN group compared to those from the delayed EN group. The improvements of total bilirubin (median [IQR]: 2.9 [-0.5-10.8] *vs*. 11.7 [5.8-25.1] μmol/L; p=0.31) and white blood cell (median [IQR]: 4.0 [0.8-6.6] *vs*. 4.4 [0.1-7.5] 10^3^/μL; p=0.72) levels were similar between the two groups. Other blood test results (urea and procalcitonin) were not included in the final analysis because of sparse data. No significant difference was observed between the two groups in the incidence of infected pancreatic necrosis (3.2% *vs*. 16.4%; p=0.096), bacteremia (0 *vs*. 10.4%; p=0.094), polymicrobial infection (16.1 *vs*. 29.8%; p=0.15), or MDRO (16.1 *vs*. 20.9%; p=0.78). The median hospital care costs per patient were $9,289 in the early EN group compared with $13,518 in the delayed EN group, in favor of the early EN group (p=0.007).

**Table 2:** Clinical Outcomes.

Outcome	Early EN (n=31)	Delayed EN (n=67)	p Value
Mortality	0	8 (11.9%)	**0.045**
Length of hospital stay, days	18 (12-30)	27 (18-43)	**0.001**
Intensive care admission	1 (3.2%)	14 (20.9%)	**0.032**
Surgical intervention	0	5 (7.5%)	0.18
Improvements of blood tests results After 7-10 days of EN			
Total bilirubin, μmol/L	2.9 (-0.5-10.8)	11.7 (5.8-25.1)	0.31
Total protein, g/L	-3.9 (-9.2-1.3)	2.1 (-5.4-6.1)	**0.03**
White blood cell, 10^3^/μL	4.0 (0.8-6.6)	4.4 (0.1-7.5)	0.72
CRP, mg/L	129.0 (63.8-177.4)	31.7 (-6.1-123.8)	**0.006**
Pancreatic necrosis	27 (87.0%)	61 (91.0%)	0.72
Infected pancreatic necrosis	1 (3.2%)	11 (16.4%)	0.096
Bacteremia	0	7 (10.4%)	0.094
Polymicrobial infection	5 (16.1%)	20 (29.8%)	0.15
MDRO	5 (16.1%)	14 (20.9%)	0.78
Hospital care costs, US$	9289 (6263-12844)	13518 (10106-20392)	**0.007**

Data expressed as n (%), or median (IQR).

The bold values indicate that there was a significant difference.

CRP, C-reactive protein; EN, enteral nutrition; MDRO, multiple drug resistant organism.

### ERCP Characteristics

The ERCP characteristics were similar between the two groups (**Table 3**). Intact papilla accounted for 96.8% of the patients in the early EN group compared with 82.1% in the delayed EN group. In the early EN group, successful biliary cannulation was achieved in 29 (93.5%) of the 31 patients, 25 of whom underwent sphincterotomy. In the delayed EN group, successful biliary cannulation was achieved in 59 (88.1%) of the 67 patients, 53 of whom underwent sphincterotomy. In all 10 patients, biliary cannulation failed because of edematous papilla. Precut sphincterotomy was performed in 6 (19.4%) of the 31 patients in the early EN group, and in 15 (22.4%) of the 31 patients in the delayed EN group. ERCP-related complications were observed in only one patient in the delayed EN group (1.5%); this complication was a post-sphincterotomy bleeding incident, and pinpoint hemostasis was achieved using a clip during ERCP.

**Table 3:** ERCP characteristics.

	Early EN (n=31)	Delayed EN (n=67)	p Value
Intact papilla, n (%)	30 (96.8)	55 (82.1)	0.06
Technical success, n (%)	29 (93.5)	59 (88.1)	0.49
Pancreatic duct cannulation (unintentional), n (%)	6 (19.4)	22 (32.8)	0.23
Sphincterotomy, n (%)	25 (80.6)	53 (79.1)	0.86
Precut sphincterotomy, n (%)	6 (19.4)	15 (22.4)	0.73
Stone extraction, n (%)	14 (45.2)	22 (32.8)	0.24
ENBD, n (%)	25 (80.6)	47 (70.1)	0.27
ERBD, n (%)	3 (9.7)	11 (16.4)	0.54
ERPD, n (%)	10 (32.3)	27 (40.3)	0.45
ERCP-related complication, n (%)	0	1 (1.5)	0.49

EN, enteral nutrition; ENBD, endoscopic nasobiliary drainage; ERBD, endoscopic retrograde biliary drainage; ERCP, endoscopic retrograde cholangiopancreatography; ERPD, endoscopic retrograde pancreatic drainage.

### Spectrum and Distribution of Pathogens

A total of 91 pathogenic bacterial strains were isolated from 60 specimens, including 47 transpapillary drainage specimens, 7 blood specimens, and 6 percutaneous drainage specimens (**Table 4**). Forty-six Gram-negative bacterial strains accounted for 50.5% of the isolates, 36 Gram-positive bacterial strains for 39.6%, and 9 fungi for 9.9%. Among the Gram-negative bacteria, the main pathogens were *Klebsiella pneumoniae*, *Stenotrophomonas maltophilia*, *Escherichia coli*, and *Enterobacter cloacae* (in descending order of infection frequency). *Enterococcus faecium*, *Enterococcus faecalis*, and *Staphylococcus* spp. were the main Gram-positive bacteria. *Candida albicans* and *Candida tropicalis* were the main fungi. Some patients had polymicrobial infections; however, no significant difference was found between the two groups (16.1% *vs*. 29.8%; p=0.15). (**Table 2**)

**Table 4:** Total microorganisms and multidrug resistant microorganisms.

	Isolates, NO. (%)
**Total microorganisms**	**91**
Total isolated GNB	46 (50.5)
* Klebsiella pneumoniae*	13 (14.3)
* Stenotrophomonas maltophilia*	10 (11.0)
* Escherichia coli*	6 (6.6)
* Enterobacter cloacae*	4 (4.4)
* Acinetobacter baumannii*	3 (3.3)
* Pseudomonas aeruginosa*	2 (2.2)
* Enterobacter aerogenes*	2 (2.2)
* Citrobacter freundii*	2 (2.2)
Others	4 (4.4)
Total isolated GPB	36 (39.6)
* Enterococcus faecium*	24 (26.4)
* Enterococcus faecalis*	6 (6.6)
* Staphylococcus aureus*	1 (1.1)
* Streptococcus viridans*	1 (1.1)
* Staphylococcus hominis*	1 (1.1)
* Staphylococcus haemolyticus*	1 (1.1)
* Staphylococcus epidermidis*	1 (1.1)
Others	1 (1.1)
Fungus	9 (9.9)
* Candida albicans*	5 (5.5)
* Candida tropicalis*	2 (2.2)
* Candida parapsilosis*	1 (1.1)
Other yeast-like fungi	1 (1.1)
**Multidrug resistant bacteria**	**25**
Total isolated multidrug resistant GNB	12 (48)
Carbapenem-resistant *Klebsiella pneumoniae*	3 (12)
ESBL-producing *Klebsiella pneumoniae*	3 (12)
ESBL-producing *Enterobacter cloacae*	1 (4)
ESBL-producing *Escherichia coli*	1 (4)
MDR *Stenotrophomonas maltophilia*	1 (4)
Carbapenem-resistant *Stenotrophomonas maltophilia*	1 (4)
Carbapenem-resistant *Pseudomonas aeruginosa*	1 (4)
Carbapenem-resistant *Klebsiella oxytoca*	1 (4)
Total isolated multidrug resistant GPB	13 (52)
MDR *Enterococcus faecium*	12 (48)
MDR Other G+ bacilli	1 (4)
**Origin of specimen**	**60**
Trans-papillary drainage	47 (78.3)
Blood	7 (11.7)
Percutaneous drainage	6 (10.0)

ESBL, extended-spectrum beta-lactamase; GNB, Gram-negative bacteria; GPB, Gram-positive bacteria; MDR, Multidrug resistant. The bold values represent sum total of isolates.

**Figure 1:**
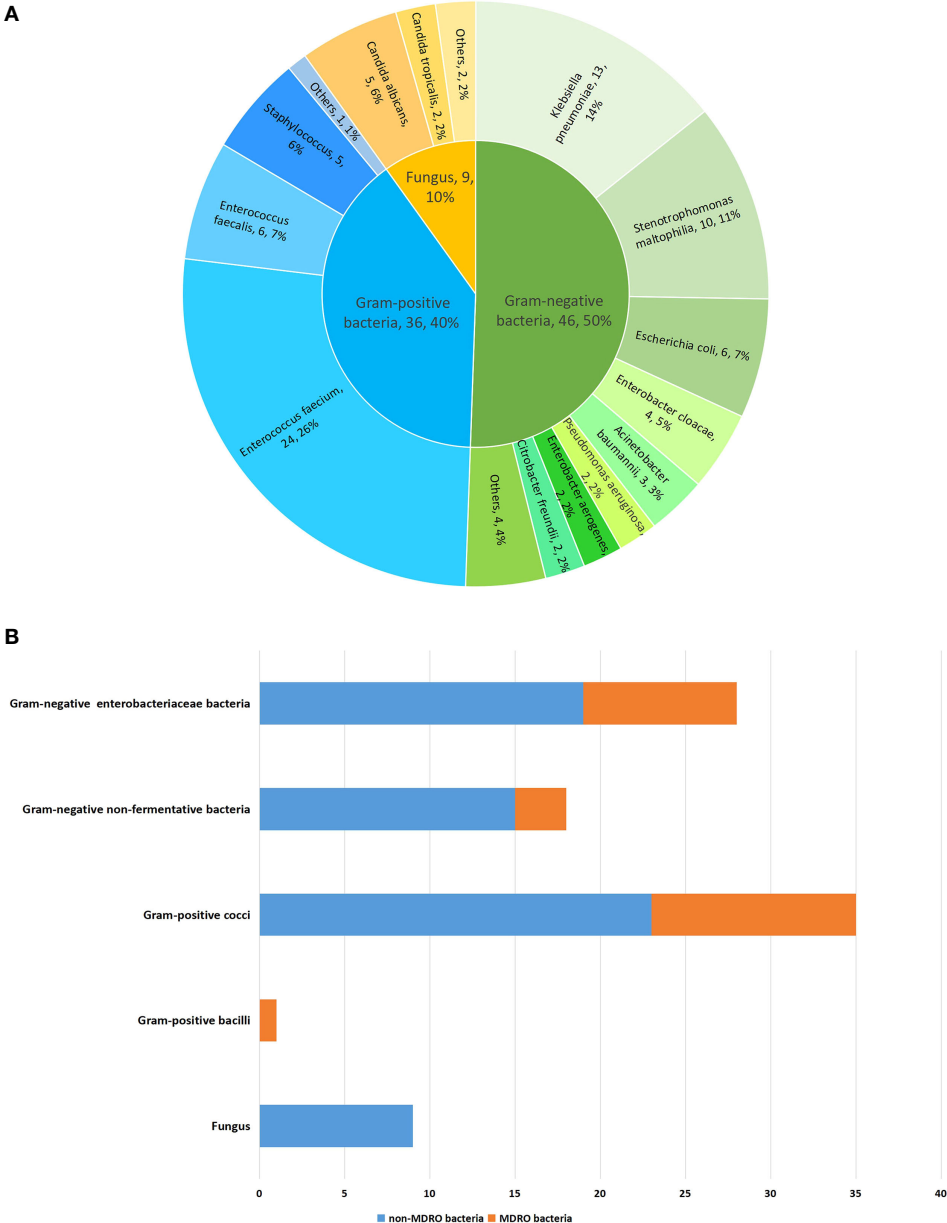
The distribution and proportion of pathogenic bacteria in 98 patients with predicted severe acute gallstone pancreatitis. **(A)** The distribution and proportion of pathogenic bacteria. **(B)** The distribution and proportion of pathogenic bacteria by dividing of MDRO.

Infection with MDRO was diagnosed in 19.4% (19/98) of patients. It occurred in 5 patients (16.1%) in the early EN group, compared to 14 (20.9%) in the delayed EN group. No significant difference was found between the two groups (p=0.78). (**Table 2**) In total, 25 isolates (27.5%) of MDRO were detected, of which 12 (48%) were MDR Gram-negative and 13 (52%) were MDR Gram-positive bacteria. No obvious drug resistance in fungi was observed. The most common MDRO was MDR *Enterococcus faecium* (n=12). Other common pathogens were carbapenem-resistant *Klebsiella pneumoniae* (n=3) and ESBL-producing *Klebsiella pneumoniae* (n=3). The distributions and proportions of the pathogenic bacteria are shown in detail in **Figure 1**.

## Discussion

The present study demonstrated that early EN in predicted SAGP was associated with a decrease in in-hospital mortality, length of hospital stay, need for intensive care admission, and hospital care costs, compared with delayed EN. In addition, early EN had more beneficial effects on blood test results than delayed EN, including total protein and CRP levels.

The fact that early EN is beneficial in AP is supported by experimental and clinical data. (Gupta et al., 2003; Yi et al., 2012) Guidelines have recommended early oral feeding in mild AP. (Tenner et al., 2013) In patients with predicted SAP, hospital stay is typically prolonged and patients are often intolerant to oral feeding. In these latter groups of patients, establishing a definite diagnosis of SAP usually occurs between 3 and 5 days after the initial presentation, a time when nasoenteral feeding is recommended to prevent infected necrosis. (Vege et al., 2018) To date, there have been few studies focusing on direct comparisons of the early and delayed timing of initiation of EN in predicted SAP.

A retrospective analysis (Wereszczynska-Siemiatkowska et al., 2013) has compared EN started within 48 h of admission to EN started 48 h after admission in SAP and revealed that early EN reduced infection of pancreatic necrosis or fluid collection, respiratory failure, mortality, and the need for intensive care admission, compared to delayed EN. However, the etiology of AP in that study was heterogeneous, including alcohol and gallstones. Whether the ERCPs performed in both study arms were comparable is unknown, and this may have caused potential bias. Nakashima et al. noted that EN initiation within 48 h after the diagnosis of SAP was associated with reduced mortality compared with late EN. Moreover, EN within 24 h may not confer more benefits than EN between 24 and 48 h. (Nakashima et al., 2021) However, none of the other clinical outcomes were evaluated.

Our study is the largest observational study to date focusing on patients with predicted SAGP who underwent ERCP. Our results are consistent with some of the data from the above-mentioned studies. These findings seem to corroborate the theory that maintaining EN modulates the acute-phase response and preserves visceral protein metabolism. (Kudsk et al., 1994) Moreover, early EN is thought to help protecting the gut mucosal barrier, thereby reducing bacterial translocation and the risk of infectious complications. (Windsor et al., 1998) However, our study did not show the superiority of early EN in reducing the rate of infection-related complications. There are several possible explanations for this negative result. First, tube feeding in the early EN group should have been started even earlier, for example, within 24 h of admission. However, in daily practice, patients with SAP are often intolerant to an earlier start of tube feeding. In our cohort, only 11 patients were administered EN within 24 h of admission, which was not sufficient to detect differences between the study arms. Second, our cohort included a large proportion of patients with cholangitis (57.1%), which could have influenced the final results associated with infection. Third, we observed that early EN was associated with a trend toward a decreased incidence of infectious complications, as shown in **Table 2**. The differences between the two groups were not significant, probably because of the small sample size. Additional studies with larger sample sizes are warranted to further investigate this.

In the early EN group in our study, a nasoenteral feeding tube was placed in the same session as the ERCP. This would relieve the suffering of patients by avoiding another step for tube placement, and would reduce the risk of repeated anesthesia, as well as hospital care costs. It’s worth noting that nasoenteral tube feeding frequently causes excessive nausea, abdominal distension, or diarrhea. Enteral nutritional fluids preheated and started at a slow rate were effective in decreasing adverse reactions in our study.

The present study indicates that infection with MDRO was found in 19.4% of the patients, which was generally lower than the level reported in previous studies. (Lee et al., 2014; Tian et al., 2020) This could be explained by the restricted use of prophylactic antibiotics in our study. MDR pathogens may be selected by unrestricted usage of broad-spectrum antibiotics. (Behrman et al., 2011) Whereas a high frequency of enteric bacilli might have been anticipated, our results were noteworthy for the high detection rate of Gram-positive bacteria in contrast to the results of a previous study. (Zhu et al., 2021) The most common MDRO in our study was MDR *Enterococcus faecium*. There are several possible explanations for this finding. First, all participants underwent ERCP; hence, duodenoscope-transmitted MDR bacterial infections should also be considered. (Balan et al., 2019) The nosocomial infections after ERCP are more polymicrobial, with a predominance of Gram-positive bacteria. Moreover, the presence of central venous catheters and abdominal drainage may also increase the risk of nosocomial infections. Second, long-term use of broad-spectrum antibiotics would alter the bacteriology of pancreatic infections in SAP from predominantly Gram-negative coliforms to predominantly Gram-positive organisms. (Howard and Temple, 2002)

The findings of this study should be interpreted in the context of its limitations. First, the results of our study were derived from a single-center experience. Therefore, the specific microbiological profiles cannot be generalized to other hospitals. Second, patients with concomitant cholangitis were included in this study; therefore, the incidence of infections due to SAP may have been overestimated, although the basis of gallstone etiology in both study arms is comparable. Third, scoring systems for the prediction of severity of AP are only moderately accurate, which could lead to the inclusion of patients who are initially classified as high risk for SAP, but who eventually develop mild pancreatitis. Fourth, the retrospective nature of the study increases the likelihood of recall and selection biases. Fifth, the sample size in the early EN group was relatively small. Thus, the interpretation of our results is limited by the difference in the sample size between the two groups; additional future comparisons with balanced data are therefore required.

## Conclusion

In conclusion, the present study demonstrates that in patients with predicted SAGP, early EN started within 48 h of admission is associated with a decrease in in-hospital mortality, length of hospital stay, need for intensive care admission, and hospital care costs, compared to delayed EN started after 48 h. However, the need for surgical intervention, and incidence of pancreatic necrosis do not differ between the two groups. In our study, Gram-negative bacteria are the main responsible pathogens. Infection with MDRO is found in 19.4% of the patients. The most common MDRO is MDR *Enterococcus faecium*. Early EN is not superior in reducing incidence of infected pancreatic necrosis, bacteremia, polymicrobial infection, and MDROs. Further studies with larger sample sizes are required to confirm our results.

## Data Availability Statement

The original contributions presented in the study are included in the article. Further inquiries can be directed to the corresponding authors.

## Ethics Statement

The studies involving human participants were reviewed and approved by ethics committee of the Hangzhou First People’s Hospital. The patients/participants provided their written informed consent to participate in this study.

## Author Contributions

HJ and XZ: study design, reviewing, and final approval; ZJ: analysis, manuscript writing, and reviewing; YW and SH: data collection and manuscript reviewing; MS, MF, and HS: data collection; JY: manuscript reviewing; All authors contributed to the article and approved the submitted version.

## Funding

The study was supported by Zhejiang Province Medical Health Science and Technology Project (Grant No: 2020KY702) and the Natural Science Foundation of Zhejiang Province (Grant No: LGF21H310004). The funder of the study had no role in the study design, data collection, data analysis, data interpretation, or writing of the article.

## Conflict of Interest

The authors declare that the research was conducted in the absence of any commercial or financial relationships that could be construed as a potential conflict of interest.

## Publisher’s Note

All claims expressed in this article are solely those of the authors and do not necessarily represent those of their affiliated organizations, or those of the publisher, the editors and the reviewers. Any product that may be evaluated in this article, or claim that may be made by its manufacturer, is not guaranteed or endorsed by the publisher.
